# Student Employment Models for Undergraduate Nurses and Midwives in Australia: A Scoping Review

**DOI:** 10.1177/23779608231186026

**Published:** 2023-07-02

**Authors:** David J. Lindsay, Tracey A. Ahern, Madelyn K. Pardon, Marie T. McAuliffe, Sam G. Rannard

**Affiliations:** 1 College of Healthcare Sciences, Nursing and Midwifery, 8001 James Cook University, Townsville, Australia; 2 College of Healthcare Sciences, Department of Psychology, 8001 James Cook University, Townsville, Australia; 3 Library and Information Services, 8001 James Cook University, Townsville, Australia

**Keywords:** midwifery students, employment, Australia, nursing students

## Abstract

**Introduction:**

Evidence has shown that throughout their undergraduate years, many nursing and midwifery students obtain paid employment in a wide variety of clinical and non-clinical positions. Across Australia, inconsistencies exist in the models of clinical employment available to these student groups. Previous Australian studies have described the employment of undergraduate nursing and midwifery students in regulated and unregulated clinical roles. No studies have reported on the various regulated roles available to both student nurses and midwives in Australia. The purpose of this scoping review is to identify and synthesize evidence related to nursing and/or midwifery students employed in regulated and unregulated clinical roles in Australia.

**Methods:**

This scoping review utilized published recommendations for data screening, abstraction, and synthesis. One of the authors, a librarian, undertook systematic searches in CINAHL Complete (1937–present), Emcare on Ovid (1995–present), Scopus (1969–present), and Ovid MEDLINE(R) (including Epub Ahead of Print, In-Process, and In-Data-Review & Other Non-Indexed Citations, 1946–present). The initial searches were completed in April 2019 and repeated in March 2021 and May 2022 to identify any new literature. Manual searching of reference lists in the included papers was also undertaken, together with selected organizational websites. The extracted data included the lead author, date, title, study design, study sample and location, and key findings.

**Results:**

From the 53 items retrieved, 23 peer-reviewed studies met the inclusion criteria and were included in the review. All items were published between 2011 and 2022. Only four of the studies focused upon student midwives. Undergraduate nursing and midwifery students in Australia obtain paid employment in a variety of regulated and unregulated clinical roles.

**Conclusion:**

The literature reported here demonstrates that there are differing models, nomenclature, educational requirements, and pay scales in place for student employment in clinical roles across Australian states and territories.

## Introduction

Globally, undergraduate nursing and midwifery students engage in paid employment as ancillary and adjunctive staff across a diverse range of clinical and residential care settings, creating an assortment of skill mixes and models of care across these sectors (Algoso et al., 2018; Mumford et al., 2022). In American and Canadian contexts, models of paid student employment in clinical roles including intern (residency) and extern programs and “cooperative partnerships,” have been in place for several decades (Friday et al., 2015; Gamroth et al., 2006; Keating et al., 1994; Rugs et al., 2020; White et al., 2019). Scholarship-based partnership models involving health service collectives and universities have also been reported in American settings (Kee & Ryser, 2001). Similarly, in countries such as the United Kingdom and New Zealand, student nurses have been employed in health-related areas while studying (Hasson et al., 2013; Mitchell, 2020). Midwifery assistant roles in the United Kingdom have also been described (McKenna et al., 2003).

In Australia, regulated roles include the Registered Undergraduate Student of Nursing (RUSON) (McGillion et al., 2022) and Registered Undergraduate Student of Midwifery (RUSOM) (Sweet et al., 2022) within the state of Victoria. A Queensland trial (Raffelt et al., 2018) (reported in this review) describes the implementation of an “Undergraduate Student in Nursing” (USiN) program at a large pediatric hospital in the capital, Brisbane.

Alongside these roles are non-professional, unregulated Personal Care Workers (PCW)/Assistants in Nursing (AIN) in the aged and disability sectors, who undertake tasks such as feeding, showering, dressing, toileting, and assisting with mobility (Schwartz, 2019). The Australian Nursing & Midwifery Federation (ANMF) has called for the regulation of this workforce under the *Health Practitioner Regulation National Law Act* 2009 (ANMF, 2019). Recommendations for consistent educational preparation and national registration have also come from the recent Royal Commission into Aged Care Quality and Safety (Commonwealth of Australia, 2021). Such roles have, in part, evolved as a result of workforce shortages, which have forced employers and regulators to explore new skill mix solutions in an era of declining nursing and midwifery workforce numbers relative to need (Algoso & Peters, 2012).

Rapid technological change and an ageing population with increasingly complex healthcare needs have also been important drivers for the development of these roles (Australian Nursing and Midwifery Federation 2021–2022), as has the financial burden associated with university study and rising cost of living expenses (Phillips et al.*,* 2012; Salamonson et al., 2020). Travel and accommodation costs associated with blocks of clinical placement, particularly in rural or remote areas, combined with lost wages while on placement, place a double financial burden upon nursing and midwifery students in Australia, which they must bear without any government support apart from means tested Centrelink payments (Grant-Smith & de Zwaan, 2019; Phillips et al., 2016; Usher et al., 2022).

The manifold effects, both positive and negative, of undergraduate students undertaking paid work while studying have been widely reported (Christiansen et al., 2019; Crawford et al., 2020; Creed et al., 2015; Grimmond et al., 2020; Robotham, 2012; Salamonson et al., 2012; Salamonson et al., 2020). The relationships between factors such as types of employment, employability following graduation, and academic success have also been described (Warner et al., 2020).

Positive aspects of such employment include greater financial independence, development of skills such as communication, time management, problem-solving, workplace socialization, and working as a member of a team (Crawford et al., 2020; Gamroth et al., 2006). Students employed in clinical environments during times of staff shortage, such as during the COVID pandemic, reportedly found the learning experience of benefit to their development as a nurse (Dempsey et al., 2023). Negative impacts include the potential for reduced academic performance, balancing employment, study and personal life, and scope of practice incongruity (Kenny et al., 2012; Mitchell, 2020).

In Australia, the tertiary-level preparation of registered nurses and midwives has been in place for many decades, and much has been written regarding the “work readiness” of nursing and midwifery graduates (Harrison et al., 2020; Missen et al., 2015; Walker et al., 2013). The ways in which practical experience gained from parttime employment, extracurricular intern and externships, and work-integrated learning (WIL) opportunities contributes to a graduate's work readiness have highlighted the potential value of such experiences for these cadres of students (Berndtsson et al., 2020; Jackson et al., 2020).

A primary rationale for this review is to better understand the differences between regulated and unregulated employment roles for nursing and midwifery students in Australia. Furthermore, there appears to be a knowledge gap regarding the employment of undergraduate nurses and midwives across the states and territories of Australia. A scoping review was thus undertaken to explore the available peer-reviewed and relevant gray literature related to this topic. This type of review was deemed appropriate given the multifaceted nature of the literature on this subject, which spans industrial, professional, educational, and workforce sectors.

## Methods

### Study Design

The scoping review procedures were guided by the methodological framework developed by Arksey and O’Malley (2005) and subsequently refined by Levac et al. (2010), Colquhoun et al. (2014), and Cooper et al. (2021). The stages include identifying the research question; identifying relevant literature; study selection; charting the data; collating, summarizing, and reporting the results; and consulting with relevant stakeholders. The PRISMA-ScR extension for scoping reviews described by Tricco et al. (2018) constituted the framework for reporting this review and is presented in Supplemental Appendix 1. Title and abstract screening, full-text review, and data extraction were undertaken by all members of the project team. Data were categorized and coded in order to chart the available evidence.

### Stage 1: Research Questions

This review was undertaken to address the following exploratory question:

1. What is known about student in nursing or student in midwifery employment roles within Australia?

### Stage 2: Identifying Relevant Literature

Searches were undertaken in the following databases: CINAHL Complete (1937–present), Emcare on Ovid (1995–present), Scopus (1969–present), and Ovid MEDLINE(R) (including Epub Ahead of Print, In-Process, and In-Data-Review & Other Non-Indexed Citations, 1946–present). The full set of Informit databases were also searched in April 2019 but was not included in subsequent searches due to database changes and loss of functionality. Retrieved articles included qualitative, quantitative, and mixed methods studies. Publicly available, relevant gray literature items were also included, such as industry reports. The latter were produced by Victorian health departments and provided valuable contextual and descriptive information.

The initial searches were completed in April 2019 and repeated in March 2021 and May 2022 to identify any new literature.

### Eligibility Criteria

Items were included if they were published research studies (primary or secondary) and focused upon models of undergraduate nursing or midwifery employment in clinical roles in Australia. Search terms included keywords listed below, combined with related MeSH terms and subject headings from other databases where appropriate. Note that a full electronic search strategy for Medline (Ovid) has been included as Supplemental material (Table 3) so that it can be repeated as required. A keyword table (Supplemental Table 4) describing which search terms correspond to MeSH terms and which are free-text terms has also been developed and included as supplemental material.

(nursing students or midwifery students) AND (hospitals OR health services OR wards OR delivery rooms) AND (personnel selection OR personnel staffing OR externships OR job experience OR paid work experience OR employment OR job OR work OR career OR work integrated learning OR rusom or ruson or registered undergraduate student of nursing or assistants in nursing or assistants in midwifery or registered undergraduate student of midwifery or sinsim or students in nursing or students in midwifery or usim or usin or assistant in nursing or assistant in midwifery). Phrase searches and truncation tools were used where applicable, and no language or date limits were applied. To capture any items not included in the initial database searches, we also hand searched the reference lists for articles included in the review. Gray literature searches were also completed using the Google site search function for the domains .gov.au, .org.au, and .asn.au. Search terms used included the following keywords and phrases: rusom or ruson or registered undergraduate student of nursing registered undergraduate students of midwifery or assistants in nursing or assistants in midwifery or registered undergraduate student of midwifery or sinsim or students in nursing or students in midwifery or usim or usin (undergraduate student in nursing, undergraduate student in midwifery). The first 100 results of each search were scanned for relevant sources and downloaded for screening.

### Stage 3: Study Selection

Search results were downloaded to EndNote and deduplicated before being exported to Excel for review. Reviewing was split between five authors who each reviewed approximately 1,500 results, ensuring all papers were checked by at least two reviewers. Three reviewers (D.L., T.A., and MM) independently screened the articles against the inclusion criteria, that is, Australian study setting, English language, and published research, theses, or industry reports, with a clear focus upon undergraduate nursing or midwifery student paid employment roles.

The sourced literature comprised 8,493 items. Of these, 3,651 were duplicates, which once removed left 4,842 articles for screening. An additional 30 papers were identified from hand searching reference lists of relevant articles, and 11 publicly accessible government documents and professional policy documents were identified.

Table 1 (Supplemental Appendix 2) describes the inclusion and exclusion criteria used to select items captured in the review. The review only included Australian literature, as there are significant differences in employment models between countries, particularly the United States, making this unrelatable to the Australian context.

### Stage 4: Charting the Data

Microsoft Excel was used to organize the data into a charting table, which included author/s, year, study location, problem under study, intervention (if relevant), study design, study methods, study sample, and study findings. Details of papers included in the review are shown in Supplemental Table 2.

### Stage 5: Collating, Summarizing, and Reporting the Results

During the initial screening stage, reviewers screened all individual results by reading the title and abstract of the article. Irrelevant articles were excluded, leaving 53 articles to be retrieved. Any questions regarding relevancy resulted in those papers being kept for further review. Finally, three reviewers (D.L., T.A., and M.P.) separately read the full text of all 53 remaining items to determine relevancy and inclusion. The authors reached consensus that 23 peer-reviewed items and two industry reports would be included in this scoping review (see Supplemental Table 2).

### Stage 6: Consultation With Stakeholders

While consultation is an optional stage under Arksey and O’Malley's original framework (2005), the methodological rigor that such consultation adds has been recognized and thus been included. The following points summarize this consultation:

- A research librarian (SR) was engaged throughout as a member of the team, to help define the parameters of the search strategy, undertake the searches of the relevant databases, and develop the PRISMA diagram.
- This review was undertaken to help inform a related study which investigated the employment of undergraduate students in nursing and midwifery across several public and private hospitals in North Queensland. In that study, information sessions were delivered by two members of the team (T.A. and D.L.) with staff at the Mater Hospital, Townsville, to present some of the quantitative and qualitative data arising from the study and respond to questions from those present. Feedback gained from these sessions guided the researchers involved in this scoping review.
- A “lunch and learn session” about this project was held for academic staff within the disciplines of nursing and midwifery at James Cook University, at which feedback about the study was gained.
- A member of the team (T.A.) presented at the Council of Deans of Nursing and Midwifery Symposium held at the Sunshine Coast, Queensland, on Tuesday, March 29, 2022. Connections were made with the supervisor of a PhD student at the University of Melbourne who is researching the RUSON role in Victoria. Further, a professional officer from the ANMF in Victoria communicated with T.A. about the implementation of the RUSON role in Victoria and the value that this role has had for both staff and students in health services where they are employed.

Engagement with these stakeholder individuals and groups further refined the research questions, enabled us to more fully appreciate the wide variety of clinical employment roles undertaken by undergraduates, and helped differentiate between regulated and unregulated positions.

## Results

A summary of the 25 items that comprised this review is provided in Table 2 (Supplemental Appendix 3), which includes the two RUSON Evaluation Reports by McGillion et al. (2022) and Kenny et al. (2019). The flow diagram (Supplemental Figure 1) summarizes the steps used to refine the search. The two Evaluation Reports cited above utilized a mixed methods approach and were focused upon pilot sites across Victoria. The report by McGillion et al. describes the implementation and evaluation of the RUSON role, piloted for 12 months across six ward areas within Western Health in Victoria. Forty-nine RUSONS comprised this pilot group. The earlier RUSON Final Report by Kenny et al. (2019) highlighted that the RUSON role was designed as a nursing workforce support strategy in rural and regional health services in Victoria. These authors subsequently published their evaluation findings as a journal article (Kenny et al., 2021), which was included in this current review. The article by Willetts et al. (2022) used a qualitative, exploratory design to obtain the perspectives of nurse leaders regarding the implementation of the RUSON nursing workforce model.

Four articles (Burns et al., 2022; McLachlan et al., 2011; Mumford et al., 2022; and Sweet et al., 2023) focused upon the role of the undergraduate student midwife. Notably, the recent article by Burns et al., which used an exploratory survey design and was conducted in New South Wales (NSW), describes an unregulated Assistant in Midwifery role. The much earlier study by McLachlan et al., which used a web-based survey administered to 47 midwives and 5 student midwives at the Royal Women's Hospital in Melbourne, describes an employment model whereby suitably prepared undergraduate midwifery students were employed in regulated positions as Division 2 nurses. The two more recent publications by Mumford et al. and Sweet et al. both describe the RUSOM model and were conducted in Victoria. The former study utilized a cross-sectional survey design, whereas in the latter, a mixed methods approach (focus groups and descriptive surveys) was applied.

The study by Crevacore et al. (2019), based in Western Australia, mapped the vocational-level “Certificate 111 in Health Services Assistant—Acute care” national qualification against the Bachelor of Nursing curriculum at a university in Western Australia.

Algoso et al. (2018) utilized a quantitative survey design applied to new nurse graduates who had worked in unregulated roles as an AIN or PCW. The only Queensland-based study was that published by Raffelt et al. (2018), which utilized a prospective observational design to describe the implementation of the USiN role at a large tertiary pediatric hospital in Brisbane, Australia, over a 12-month period.

A number of articles described students working in unregulated roles as (Health) AIN in a variety of clinical contexts, including aged care.

The study by Lokmic-Tomkins et al. (2022) was the only one in which graduate entry to nursing practice (Master's pathway) students employed as health assistants during their studies constituted the participant group. All other studies focused upon undergraduate students. Algoso et al. (2019; 2018) reported on undergraduate students working as nursing assistants in aged care, while others focused upon acute care environments (Crevacore et al., 2019), including the emergency department (Gerace et al., 2018), and inpatient mental health settings (Browne et al., 2013; Cleary et al., 2012). Two articles (Phillips et al., 2016; Wise et al., 2022) each provided a review of the literature regarding paid employment of undergraduate nursing students.

## Discussion

To our knowledge, this review is the first to have captured and synthesized Australian peer-reviewed and gray literature describing undergraduate nursing and midwifery employment roles. Our findings demonstrate that there is a growing body of evidence describing the various unregulated employment roles of undergraduate students in nursing; however, few studies describe models of employment that are specifically designed for student nurses and fewer still for midwifery students. Notably, only two states of Australia appear to have dedicated employment roles for undergraduate students of nursing and midwifery: the RUSON and RUSOM roles in Victoria and the USiN and USiM roles in Queensland. The RUSON/RUSOM roles are well described (McGillion et al., 2022; Sweet et al., 2022; Victoria Government, 2022; Willetts et al., 2022) and their employment parameters including rates of pay outlined within the current Victorian Public Sector Enterprise Agreement (2020–2024). Under this agreement, any period of service working in RUSON/RUSOM roles contributes to their continuity of service within the Victorian Public sector, provided the gap is <12 months between employment as a student and commencement of employment as a Registered Nurse/Registered Midwife. By contrast, the only study that described an USiN program in Queensland was that by Raffelt et al. (2018), which reported that their introduction at a large tertiary pediatric hospital had an “overwhelmingly positive affect [sic] for parents and children” (p.22). Despite the absence of a supporting policy framework when compared with those describing RUSON/RUSOM roles in Victoria, Queensland has implemented paid USiN and USiM) positions across both public and private health services in urban and regional health centers. Akin to the RUSON and RUSOM roles in Victoria described above, these “student-only” roles have *not* been explicitly developed in partnership with the students’ university, and there is no formal relationship between their employment and their respective undergraduate program. The university also does not contribute to the selection, supervision, or evaluation of students in such roles.

An examination of publicly available gray literature, such as professional nursing and midwifery organization policy documents (see, e.g., Australian Nursing & Midwifery Federation, 2020; NSW Nurses & Midwives’ Association, 2020), shows that across the various states and territories of Australia, undergraduate students in nursing and midwifery can obtain paid employment in a variety of ancillary and direct care roles in public and private hospitals. As mentioned, these roles include AIN, PCW, health assistants in nursing, SiN, or SiM in Queensland or RUSON/graduate entry to practice Master’s students (RUSON)/RUSOM in Victoria, Australia (Lokmic-Tomkins et al., 2022; McNally & Blay, 2018; Nursing and Midwifery Board of Australia [NMBA], 2020; Twigg et al., 2016). Despite the different nomenclature used to describe these roles (Duffield et al., 2014), there are several broad similarities in their employment arrangements, supervisory relationships, and remuneration. For example, as Lokmic-Tomkins et al. (2022) indicate, common characteristics are that they all require supervision by a registered nurse or registered midwife, their clinical hours do not replace or contribute to the formal clinical placement requirements within their respective degree, and their remuneration rates are protected by either an industrial award or enterprise agreement. An important distinction between some of these roles, which is reflected in their titles, is that several are designed to *only* be for bachelor-level students in nursing or midwifery; eligible students can apply for *any* of the other ancillary roles. Importantly, the use of the “ancillary” and “adjunct” descriptors for the majority of these roles means that they are not intended to be substitutive but act as support workers for the registered and enrolled nurses and midwives who are rostered within a variety of healthcare settings across metropolitan, regional, and rural areas. As Roche et al. (2016), Crevacore et al. (2019), Blay and Roche (2020), and Roche et al. (2021) note, the number of unlicensed/unregulated nurses employed in the Australian healthcare sector as AIN has dramatically increased over the past two decades, with growing numbers present across community, acute care, and mental health inpatient settings. These reports highlight the impact that these roles have had upon patient safety and describe myriad complexities associated with the integration and supervision of this cadre of unregulated workers into the staff skill mix across a range of hospital and community contexts.

All undergraduate nursing and midwifery students in Australia are registered with the Australian Health Practitioner Regulation Agency (AHPRA) and are thus subject to the relevant provisions under the National Law covering Health Practitioner Regulation (Parliament of Queensland, 2022). These provisions would apply in whatever paid health worker position the student is employed. Conversely, non-students working in unregulated roles are not registered with AHPRA and thus work outside the National Law. As Crevacore identifies in her doctoral thesis on the topic of delegation practices between registered nurses and AINs in Western Australia (2021), there is not a standard educational requirement for these roles nor do they have professional practice standards or a regulated scope of practice. These concerns are echoed in the recent independent review into nursing education (Schwartz, 2019) which, *inter alia*, recommended that “To protect the public, assistants in nursing (whatever their job title) should have mandated education, English language, and probity requirements, which are accredited, assessed, and enforced by a robust quality-assurance regime.” (p. 27). To date, this recommendation has not been taken up and what is currently in place is a National Code of Conduct for Health Care Workers (Queensland) (Council of Australian Governments, 2015), which falls well short of the regulatory requirements outlined above.

As mentioned, in the states of Victoria, NSW, and Queensland, second- or third-year Bachelor of Nursing students can be employed in dedicated student-only roles, though no literature could be found specifically describing these roles in the NSW context. As stated previously, in Victoria, these are titled as RUSON (Kenny et al., 2021; Kenny et al., 2019; Victoria Government, 2020). This latter role was specifically developed to “build workforce capacity in regional/rural health services” in that state (Kenny et al., 2019, p. 3). Similarly, midwifery students who have successfully completed at least 12 months of an accredited Bachelor of Midwifery or at least 2 years of a dual nursing and midwifery degree are able to apply for RUSOM positions in Victoria (Sweet et al., 2022). Dual-degree students can apply for either RUSON or RUSOM positions. Importantly, the RUSOM direct care role is *not* supernumerary, and it does not replace clinical practicum requirements prescribed within accreditation agreements between universities and the Australian Nursing & Midwifery Accreditation Council. The government of Victoria has published a range of documents associated with these two roles, including core duties and exclusion lists, position description templates (Victoria State Government, 2022), and a RUSON employment and implementation guide (Victoria State Government, 2020).

During the COVID-19 pandemic, RUSONs and RUSOMs were identified by the Victoria government as part of the surge workforce to undertake screening and administer vaccinations (Victoria State Government, n.d.). The Victorian branch of the ANMF’s website (https://www.anmfvic.asn.au/ruson) provides further detail regarding the RUSON COVID-19 surge workforce employment model for organizations and students.

## Conclusion

The evidence presented in this scoping review demonstrates that undergraduate nurses and midwives actively seek paid employment in clinical roles, not only for financial reasons but also to increase their exposure to these environments, develop their nursing and midwifery knowledge and skills, and enhance their opportunities for employment following graduation. Also highlighted was the wide variety of clinically oriented employment roles available to undergraduate nurses and midwives in Australia. Predominantly, PCW/AIN roles have been in place for decades as an unregulated, non-professional workforce, with varying scopes of practice and employment arrangements, different pay scales, diverse titles, and dissimilar educational preparation. Several reports described regulated roles created specifically for undergraduate nurses and midwives, such as the RUSON/RUSOM in Victoria and the USIN/USIM in Queensland. Notably, there is a paucity of literature describing or evaluating the role of student midwives in specific clinical employment roles. Few Australian studies described the regulated positions specifically created for student nurses, and only three recent reports explored the paid employment of student midwives as assistants in midwifery settings. No studies were found which referred to the relationship between undergraduate nursing and midwifery students, their university, and paid employment roles in either hospital or community settings. A more consistent, national approach to the employment of undergraduate nurses and midwives across the spectrum of healthcare settings is thus clearly needed.

### Implications for Governance, Education, Research, Policy, and Practice

There is substantial heterogeneity present within undergraduate nursing and midwifery employment models across Australia. This has created significant governance concerns that employers and industrial and professional bodies have as yet been unable to successfully address. Calls for a clearer, more consistent, national policy direction in relation to the regulation of nurses in ancillary roles have become more strident in recent years. The recent Royal Commission into Aged Care Quality and Safety in Australia, together with the COVID-19 pandemic, has focused the spotlight on this long-standing and anomalous workforce situation in that sector. Strong leadership and advocacy are required from our professional bodies and policymakers to establish unambiguous, consistent, and coherent national guidelines and structures for the education, employment, regulation, and registration of this burgeoning workforce. Moreover, the increasing competition for clinical placement positions indicates that universities must collaborate more closely with clinical facilities regarding the employment of undergraduate nursing and midwifery students, in order to recognize and incorporate their WIL experiences.

At the practice level, while the employment of students in these ancillary roles has demonstrated positive outcomes for both the student and the facility, their clinical supervision by an already stressed workforce has implications for the safety and sustainability of such roles. Robust longitudinal studies examining the strengths and weaknesses of models of undergraduate nursing and midwifery employment across all Australian states and territories would provide valuable insights into these cadres of increasingly important ancillary healthcare workers. Appropriately funded empirical research that will generate the requisite evidence is desperately needed if we are to move forward in a manner which reflects our accountability to the public we serve.

### Limitations

The authors recognize that a number of the studies included in this review demonstrated significant bias, which must be taken into account when considering the reliability and generalizability of the stated results. Given that formal assessments of risk of bias for each included study were not presented, the authors acknowledge that this also constitutes a limitation of this review. Access to other databases may have produced a more robust sample, and the incorporation of industry-based literature relied upon its availability in the public domain. The focus of this review was upon Australian literature; thus, no comparison was made with other countries regarding the diversity of undergraduate nursing and midwifery employment roles and relationships.

## Supplemental Material

sj-docx-1-son-10.1177_23779608231186026 - Supplemental material for Student Employment Models for Undergraduate Nurses and Midwives in Australia: A Scoping Review Click here for additional data file. Supplemental material, sj-docx-1-son-10.1177_23779608231186026 for Student Employment Models for Undergraduate Nurses and Midwives in Australia: A Scoping Review by David J. Lindsay, Tracey A. Ahern, Madelyn K. Pardon, Marie T. McAuliffe and Sam G. Rannard in SAGE Open Nursing

sj-docx-2-son-10.1177_23779608231186026 - Supplemental material for Student Employment Models for Undergraduate Nurses and Midwives in Australia: A Scoping Review Click here for additional data file. Supplemental material, sj-docx-2-son-10.1177_23779608231186026 for Student Employment Models for Undergraduate Nurses and Midwives in Australia: A Scoping Review by David J. Lindsay, Tracey A. Ahern, Madelyn K. Pardon, Marie T. McAuliffe and Sam G. Rannard in SAGE Open Nursing

sj-docx-3-son-10.1177_23779608231186026 - Supplemental material for Student Employment Models for Undergraduate Nurses and Midwives in Australia: A Scoping Review Click here for additional data file. Supplemental material, sj-docx-3-son-10.1177_23779608231186026 for Student Employment Models for Undergraduate Nurses and Midwives in Australia: A Scoping Review by David J. Lindsay, Tracey A. Ahern, Madelyn K. Pardon, Marie T. McAuliffe and Sam G. Rannard in SAGE Open Nursing

sj-docx-4-son-10.1177_23779608231186026 - Supplemental material for Student Employment Models for Undergraduate Nurses and Midwives in Australia: A Scoping Review Click here for additional data file. Supplemental material, sj-docx-4-son-10.1177_23779608231186026 for Student Employment Models for Undergraduate Nurses and Midwives in Australia: A Scoping Review by David J. Lindsay, Tracey A. Ahern, Madelyn K. Pardon, Marie T. McAuliffe and Sam G. Rannard in SAGE Open Nursing

sj-docx-5-son-10.1177_23779608231186026 - Supplemental material for Student Employment Models for Undergraduate Nurses and Midwives in Australia: A Scoping Review Click here for additional data file. Supplemental material, sj-docx-5-son-10.1177_23779608231186026 for Student Employment Models for Undergraduate Nurses and Midwives in Australia: A Scoping Review by David J. Lindsay, Tracey A. Ahern, Madelyn K. Pardon, Marie T. McAuliffe and Sam G. Rannard in SAGE Open Nursing
