# Supplementary material for: Student Employment Models for Undergraduate Nurses and Midwives in Australia: A Scoping Review
Source: SAGE Open Nurs. 2023 Jul 2;9:23779608231186026. doi: 10.1177/23779608231186026 (PMC10328162; doi:10.1177/23779608231186026)
Supplement: sj-docx-1-son-10.1177_23779608231186026 - Supplemental material for Student Employment Models for Undergraduate Nurses and Midwives in Australia: A Scoping Review [file sj-docx-1-son-10.1177_23779608231186026.docx]

**Figure 1: PRISMA 2020 flow diagram for new systematic reviews which included searches of databases, registers and other sources**

**Identification of studies via other methods**

**Identification of studies via databases and registers**

Records identified from:

Organisations (n = 11)

Citation searching (n = 30)

Records removed *before screening*:

Duplicate records removed (n = 3651)

Records identified from: Databases (n = 8493)

**Identification**

Records screened

(n = 4842)

Records excluded

(n = 4789)

Reports not retrieved

(n = 0)

Reports sought for retrieval

(n = 41)

Reports sought for retrieval

(n = 53)

Reports not retrieved

(n = 0)

**Screening**

Reports excluded:

Non-Australian (n = 18)

Unpaid placement (n = 1)

No original research (n = 6)

Non-student positions (n = 2)

Reports excluded:

Non-Australian (n = 35)

Non-health related employment (n = 1)

Unpaid placement (n = 1)

Residency placement (n = 1)

Reports assessed for eligibility

(n = 41)

Reports assessed for eligibility

(n = 53)

Studies included in review

(n = 25

Reports of included studies

(n = 0)

**Included**

*From:*  Page MJ, McKenzie JE, Bossuyt PM, Boutron I, Hoffmann TC, Mulrow CD, et al. The PRISMA 2020 statement: an updated guideline for reporting systematic reviews. BMJ 2021;372:n71. doi: 10.1136/bmj.n71. For more information, visit: <http://www.prisma-statement.org/>
