# Supplementary material for: Student Employment Models for Undergraduate Nurses and Midwives in Australia: A Scoping Review
Source: SAGE Open Nurs. 2023 Jul 2;9:23779608231186026. doi: 10.1177/23779608231186026 (PMC10328162; doi:10.1177/23779608231186026)
Supplement: sj-docx-2-son-10.1177_23779608231186026 - Supplemental material for Student Employment Models for Undergraduate Nurses and Midwives in Australia: A Scoping Review [file sj-docx-2-son-10.1177_23779608231186026.docx]

**Table 1: Inclusion and exclusion criteria used for selecting articles**

| Criteria | Inclusion | Exclusion |
| --- | --- | --- |
| Location | Australian studies | Non-Australian studies |
| Dates | No limits set |  |
| Language | English | Non-English |
| Population and sample | Undergraduate nursing or midwifery students enrolled in a recognised program | All other nurses, health care workers or students not enrolled in a recognised undergraduate program |
| Type of source | Original research, published in a peer reviewed journal  Grey literature | Non-peer reviewed sources  Not grey literature |
| Ethics clearance | Studies with approved ethics notiﬁcation | No ethics approval |
| Study focus | Undergraduate student nurses or midwives working in student nursing roles | All other nurses, all other students, all other employment types |
| Literature focus | Articles where overwhelming theme relates to undergraduate student nurse or midwife employment | Passing or token reference to U/G student employment |
