# Supplementary material for: Student Employment Models for Undergraduate Nurses and Midwives in Australia: A Scoping Review
Source: SAGE Open Nurs. 2023 Jul 2;9:23779608231186026. doi: 10.1177/23779608231186026 (PMC10328162; doi:10.1177/23779608231186026)
Supplement: sj-docx-3-son-10.1177_23779608231186026 - Supplemental material for Student Employment Models for Undergraduate Nurses and Midwives in Australia: A Scoping Review [file sj-docx-3-son-10.1177_23779608231186026.docx]

**Table 2 Summarized Findings of Included Articles**

| Lead author (year) | Title | Study Design | Sample and location of study | Results |
| --- | --- | --- | --- | --- |
| McGillion et al. (2022) | An Evaluation of the RUSON Pilot at Western Health. | Mixed method design. | Pilot of RUSONs (n=45) was conducted from November 2020 to November 2021 in six ward areas of Western Health, Victoria. | Specific benefits to the health service and workforce were found, prompting expansion of the RUSON workforce at Western Health. Recommendation for more structured induction processes and scope of practice guidance. |
| Burns et al. (2022) | Hospital-based assistant in Midwifery role for undergraduate midwifery students: A survey exploration. | Exploratory survey design. | 128 undergraduate midwifery students employed as Assistants in Midwifery (AIM) in New South Wales, Australia over a five-year period. Analysis of position descriptions for all AIM jobs advertised between September 2019 and February 2020. | AIM job descriptions need to be specific to the profession of midwifery. |
| Mumford et al. (2022) | Supporting the midwifery workforce: An evaluation of an undergraduate midwifery student employment model at a large tertiary maternity service in Victoria, Australia. | Cross-sectional survey design (predominantly quantitative, with some open questions). | 20 RUSOMs and 115 midwifery staff, in a public tertiary maternity hospital in Melbourne, Australia. | RUSOM model found to be a positive workforce strategy, increasing work readiness, confidence and competence to practise. |
| Lokmic-Tomkins et al. (2022) | Improving the health assistant in nursing employment model through entry to practice nursing student perceptions: a cross-section study. | Descriptive, qualitative study. | 38 entry to practice nursing students (Master’s pathway) employed as health assistants in nursing (HANs) in one university hospital in Melbourne, Australia. | HAN model provided benefits in terms of patient satisfaction, competence and confidence. Scope of practice found to be too limited. |
| Sweet et al. (2022) | The introduction of registered undergraduate students of midwifery in a tertiary hospital: Experiences of staff, supervisors, and women. | Mixed method design. | Nine focus groups (n=41) and four descriptive surveys (n=135) were used to collect data from RUSOM staff, maternity and medical staff, and women who received care from a RUSOM in a postnatal ward in a large maternity hospital in Australia. | Findings demonstrate benefits for undergraduate midwifery students as well as the women and the organisation. Study supports the expansion of the RUSOM model in Australia. |
| Wise et al. (2022) | The contribution of paid employment for students during their pre-registration nursing studies: An integrative review. | An integrative review. | 14 studies (both Australian and international) included in the integrative review. | Identification and description of four key attributes associated with building workplace capacity that pre-registration nursing students develop through paid employment whilst undertaking their studies. |
| Kenny et al. (2021) | Evaluation of rural undergraduate nursing student employment model. | Mixed method  Interviews, focus groups and blog data were used to collect qualitative data. A survey was used to collect quantitative data. | Purposive sampling included Registered Undergraduate Students of Nursing (RUSONs) (N=21), as well as staff and patients/clients across 12 rural health services in Victoria. | Paid employment models for students of benefit in rural health services. More rigorous evaluation of these models is required. Across Australia, Government interest in the role appears highly variable. |
| Willetts et al. (2021) | Implementation of Registered Undergraduate Student of Nursing (RUSON) program: The nurses’ perspective. | Qualitative exploratory design. | A purposive sample of 6 nurse leaders from the units who utilised the RUSON workforce in an oncology hospital in Australia. | RUSON model a successful workforce strategy. Successful implementation involves engagement with nursing leaders. |
| Crawford et al. (2020) | First year nursing students’ perceptions of learning interpersonal communication skills in their paid work: A multi-site Australasian study. | Qualitative exploratory design. | Fifty, first year nursing students from four higher education institutions in Australia and New Zealand. | Two primary themes identified: recognizing the value of learning interpersonal skills, and opportunities to develop effective interpersonal communication skills. Acquiring skills such as emotion management, active listening and being empathic, and multi-tasking while communicating were also identified by participants as transferrable to their nursing practice. |
| Kenny et al. (2019) | Final Report: Evaluation of Registered Undergraduate Student of Nursing (RUSON) Pilot Program. | Mixed method approach with partially mixed, concurrent, equal status design. | Extensive data collection through a range of methods to collect data from staff, RUSONs and clients to evaluate the RUSON (n=21 RUSONs) pilot program in Victoria. | Independent evaluation of the RUSON pilot confirmed most of the Department of Health and Human Services (DHHS) outcomes/hypothesis. |
| Algoso et al. (2019) | An exploration of undergraduate nursing assistant employment in aged care and its value to undergraduate nursing education. | Qualitative, retrospective design as part of a larger mixed methods study. | Responses were collected from 110 surveys from graduate RNs who had previously worked in undergraduate AIN employment in Australia. | Undergraduate AIN employment in aged care prepared novice nurses for their graduate nurse practice in areas such as higher-order thinking, communication, time management, understanding the patient perspective, the organisation and the profession. |
| Crevacore et al. (2019) | Undergraduate registered nursing students working as assistants in nursing within the acute care environment: Program development and discussion. | Discussion paper. | Discussion of the process used to offer students a VET qualification through partial completion of their undergraduate degree enabling them to work safely as AINs and seek opportunities for undergraduate RN student employment. Project was undertaken in Western Australia. | Improved AIN qualifications and work opportunities through use of the nationally endorsed Certificate III in Health Services Assistant – Acute Care qualification for undergraduate nursing students. |
| Algoso et al. (2018) | Undergraduate nursing assistant employment in aged care has benefits for new graduates. | Quantitative, descriptive design as part of a larger mixed methods study. | Responses were collected from 110 surveys from graduate RNs who had previously worked in undergraduate AIN employment in Australia. | Novice, undergraduate nurses can develop clinical skills and nursing qualities when undertaking paid employment in the aged care sector, improving preparedness for work as a graduate RN. |
| Gerace et al. (2018) | Assistants in nursing working with mental health consumers in the emergency department. | Qualitative, exploratory design. | 24 participants (AINs, Nurses, NUMs). AINs (n=8) were all studying an undergraduate nursing programme, working with mental health consumers in an ED in a South Australian hospital. | AINs were important members of the healthcare team, facilitating more positive patient experiences for consumers on involuntary mental health treatment orders in an emergency department. |
| Raffelt et al. (2018) | Incorporating an Undergraduate Student in Nursing program in the workforce: a prospective observational study. | Prospective, observational design. | Participants in pre-implementation survey (N=64), and in 12-month evaluation survey (N=105). Participants were registered nurses employed in medical, surgical, rehabilitation, pediatric intensive care and perioperative at a large tertiary pediatric hospital in Brisbane, Australia. | Positive impact on parent and child healthcare experience. Benefits also found for RN’s allowing them more time to practice higher level tasks relevant to the RN scope of practice. |
| Crevacore et al. (2016) | The effect of an enrolled nursing registration pathway program on undergraduate nursing students’ confidence level: A pre- and post-test study. | Quasi-experimental, prospective observational cohort study. | Purposive sample of 147 second and third year undergraduate nursing students at a university in Western Australia. | Students enrolled in the Enrolled Nursing Registration Pathway Program (ENRPP) were involved in meaningful employment, increasing exposure to the healthcare environment and increasing confidence in four of five domains investigated. |
| Phillips et al. (2016) | Pre-registration paid employment practices of undergraduate nursing students: A scoping review. | Scoping Review. | 40 studies were found to fit the search criteria between 1995-2013. | Many undergraduate nursing students seek paid employment in clinical settings to increase their generic skill sets in preparation for transition to RN practice. |
| Phillips et al. (2014) | Does the choice of pre-registration paid employment impact on graduate nurse transition: An Australian study. | Qualitative, exploratory study. | An Australian study consisting of data from focus groups with (n=67) new graduates, and qualitative open-ended questionnaire (n=392). | Results presented in three main themes: drawing on prior learning, the burden of employment choice, and outside the setting. Findings reveal a range of benefits and limitations to transition to RN resulting from paid employment in healthcare settings whilst studying an undergraduate nursing degree. |
| Browne et al. (2013) | Addressing the mental health nurse shortage: Undergraduate nursing students working as assistants in nursing in inpatient mental health settings. | Opinion piece. | Discussion proposes employing undergraduate nursing students as AINs in mental health settings to increase student consideration of mental health nursing as a career. | Shortages of mental health nurses and strategies to give undergraduate nursing students exposure to the mental health workforce via paid AIN positions. Positive undergraduate experiences of working as AINs in mental health can potentially attract them once they graduate. Caution should be taken to ensure AINs do not diminish the vital role of RNs in mental health settings. |
| Algoso and Peters (2012) | The experiences of undergraduate Assistants in Nursing (AIN). | Qualitative study using semi-structured interviews. | Six nursing students at a major university in New South Wales, Australia, who were employed as undergraduate AINs. | Three main themes emerged: Becoming part of the team, Understanding the scope of the AIN role, and Working outside your comfort zone. Experiences of AINs demonstrated increased confidence and exposure to the realities of nursing. |
| Cleary et al. (2012) | Promoting mental health nursing: Employing undergraduate nursing students as assistants in mental health. | Discussion piece. | Nine factors discussed that contribute to the motivation of AINs to work in mental health nursing. | Employment of undergraduate students as AINs in mental health settings should be undertaken using a strategic approach to meet the recruitment needs of employers and the educational and monetary needs of nursing students. |
| Kenny et al. (2012) | Models of nursing student employment: An Australian action research study. | Action research study. | Three phase action research study conducted in Victoria, Australia to consider explicit nursing student paid employment and develop recommendations to guide policy and planning. | Study reported the potential to improve university and practice linkages through undergraduate student paid employment programs, potentially strengthening the links between theory and practice. |
| Phillips et al. (2012) | Pre-registration paid employment choice: The views of newly qualified nurses. | Interpretive, descriptive design. | This South Australian study comprised eight focus groups including first year RNs (n=67) to derive retrospective data about pre-registration paid employment. | The majority of participants sought employment in health settings, believing this was beneficial for their career as an RN and securing employment after graduation. |
| Salamonson et al. (2012) | The impact of term-time paid work on academic performance in nursing  students: A longitudinal study. | Descriptive correlational study with longitudinal follow-up. | The sample were undergraduate nursing students in Australia. First year nursing students (n=566) participated in the baseline survey, and in their third year (n=183) students completed the follow-up survey. | New models of undergraduate nursing education that include faculty approved nursing-related employment should be explored to accommodate balancing paid work with full-time study, in an effort to mitigate academic underperformance. |
| McLachlan et al. (2011) | Addressing the midwifery workforce crisis: Evaluating an employment model for undergraduate midwifery students at a tertiary maternity hospital in Melbourne, Australia. | Quantitative, descriptive study, with survey design. | Forty-seven midwives and five undergraduate midwifery students employed as Division 2 nurses (SMW_Div2) (enrolled nurses) who worked in postnatal areas of a Victorian hospital. | The employment model for SMW_Div2s is a positive initiative for both the midwives and the SMW_Div2s, potentially offering short- and long-term advantages to the midwifery profession. |
