# Supplementary material for: Student Employment Models for Undergraduate Nurses and Midwives in Australia: A Scoping Review
Source: SAGE Open Nurs. 2023 Jul 2;9:23779608231186026. doi: 10.1177/23779608231186026 (PMC10328162; doi:10.1177/23779608231186026)
Supplement: sj-docx-4-son-10.1177_23779608231186026 - Supplemental material for Student Employment Models for Undergraduate Nurses and Midwives in Australia: A Scoping Review [file sj-docx-4-son-10.1177_23779608231186026.docx]

**Table 3: Full electronic search strategy used for Medline (Ovid)**

| Ovid MEDLINE(R) and Epub Ahead of Print, In-Process, In-Data-Review & Other Non-Indexed Citations, Daily and Versions |
| --- |
| 1 delivery rooms/ or exp hospitals/ |
| 2 (hospital* or health services or ward*).mp. |
| 3 1 or 2 |
| 4 Students, Nursing/ |
| 5 Nurse Midwifery student* or nursing student* or midwifery student*).mp. |
| 6 4 or 5 |
| 7 ("assistant* in nursing" or "assistant* in midwifery" or ruson or rusom or "registered undergraduate student of nursing" or sinsim or "students in nursing" or "students in midwifery" or usim or usin or externship* or job experience or paid work experience or work integrated learning or employment or job or work* or career).mp. |
| 8 employment/ or workplace/ |
| 9 personnel selection/ or exp "personnel staffing and scheduling"/ |
| 10 exp Workforce/ |
| 11 7 or 8 or 9 or |
| 12 3 and 6 and 11 |
