# Supplementary material for: Student Employment Models for Undergraduate Nurses and Midwives in Australia: A Scoping Review
Source: SAGE Open Nurs. 2023 Jul 2;9:23779608231186026. doi: 10.1177/23779608231186026 (PMC10328162; doi:10.1177/23779608231186026)
Supplement: sj-docx-5-son-10.1177_23779608231186026 - Supplemental material for Student Employment Models for Undergraduate Nurses and Midwives in Australia: A Scoping Review [file sj-docx-5-son-10.1177_23779608231186026.docx]

**Table 4: Search terms corresponding to MeSH terms and free-text terms**

| **Keywords (applied to all database searches)** | **Medline** | **CINAHL Subject Headings** | **Emcare** |
| --- | --- | --- | --- |
| hospital* OR health services OR ward* | delivery rooms/ OR exp hospitals/ | exp hospitals+ | delivery room/ OR hospital/ |
| nurse midwifery student* OR nursing student* OR midwifery student* | students, nursing/ | students, midwifery OR students, nurse midwifery OR students, nursing OR students, nursing, associate OR students, nursing, baccalaureate OR students, nursing, diploma programs | nursing student/ OR baccalaureate nursing student/ OR midwifery student/ |
| “assistant* in nursing" OR "assistant* in midwifery" OR ruson OR rusom OR "registered undergraduate student of nursing" OR sinsim OR "students in nursing" OR "students in midwifery" OR usim OR usin OR externship* OR job experience OR paid work experience OR work integrated learning OR employment OR job OR work* OR career | employment/ OR workplace/ OR personnel selection/ OR exp "personnel staffing and scheduling"/ OR exp workforce/ | employment OR part time employment OR temporary employment OR  workforce OR personnel staffing and scheduling OR personnel management OR personnel selection | employment/ OR parttime employment/ OR temporary employment/ OR workplace/ OR exp workforce/ OR health care personnel management/ OR hospital personnel management/ OR personnel management/ |

Medline & Emcare exp = exploded

CINAHL + = exploded
